# Health-related quality of life of patients with multidrug-resistant tuberculosis in Yemen: prospective study

**DOI:** 10.1186/s12955-019-1211-0

**Published:** 2019-08-16

**Authors:** Ammar Ali Saleh Jaber, Baharudin Ibrahim

**Affiliations:** 1 Department of Clinical Pharmacy, Faculty of Pharmacy, Universiti Sains Malaysia, 11800 Penang, Malaysia; 2 Department of Clinical Pharmacy, Faculty of Pharmacy, Taiz University, Taiz, Yemen

**Keywords:** Tuberculosis, Yemen, Health related quality of life

## Abstract

**Background:**

Substantial efforts are currently focused on investigating and developing new multidrug-resistant tuberculosis (MDR-TB) drugs and diagnostic methods. In Yemen, however, the evaluation of health-related quality of life (HRQoL) and the effect of current MDR-TB treatment on the QoL are commonly ignored. This study evaluated the HRQoL during and after treatment and identified the risk factors that are predictive of HRQoL score differences.

**Method:**

A prospective cohort study was conducted in four of the five main MDR-TB centres in Yemen. The patients confirmed with MDR-TB completed the SF-36 V2 survey at the beginning of treatment, end of treatment (continous phase) and at the 1 year follow-up after completing treatment. A total normal base score (NBS) of < 47 reflects impairment of functions, whereas a mental component summary (MCS) score of < 43 indicates a risk of depression.

**Result:**

At the beginning of treatment, the mean scores for all health domains were < 47 NBS points (PF = 40.7, RP = 16.1, BP = 21.6, GH = 28.3, VT = 14.55, SF = 25.9, RE = 13.7, and MH = 14.7). At the completion of treatment, all eight health domains increase compare to beginning of treatment (PF = 59.3, RP = 31.1, BP = 40.9, GH = 48.5, VT = 30.5, SF = 46.6, RE =26.6 & MH = 27.7), but a follow-up duration of 1 year after completing treatment showed decreased NBS points in all domains (PF = 51.5, RP = 30.6, BP = 39.1, GH = 47.8, VT = 30.2, SF = 43.7, RE =26.4 & MH = 27.2). Age, history of streptomycin use, baseline lung cavity, marital status and length of sickness before MDR-TB diagnosis were predictive of in PCS score differences, whereas, age, smoking, baseline lung cavity, stigma, residence, marital status and length of sickness before MDR-TB diagnosis were predictive of MCS scores differences.

**Conclusion:**

The length of sickness before DR-TB diagnosis was found to be predictive of the trends in both PCS and MCS scores. Despite the positive outcome of MDR-TB treatment, the low HRQoL scores obtained for all heath domains and especially for mental health reflect a high depression status of patients even after 1 year of completing therapy. Moreover, the poor HRQoL, particularly regarding mental health, of study participants at the end of treatment demands the need for urgent attention from national tuberculosis control programme managers. Therefore, the Yemen Ministry of Health and the National Tuberculosis Control Programme should implement an intervention programme to enhance HRQoL at the end of treatment to avoid any further negative consequences of MDRTB in patients after treatment. Moreover, The HRQoL data of patients with MDR-TB must be collected at the different stages of MDR-TB treatment to provide an additional parameter for assessing the effectiveness of the treatment programme.

**Trial registration:**

SNOYEM 1452. Registered 01 February 2013.

## Background

Tuberculosis (TB) is consider as one of the most transmittable disease, causing approximately 2 million deaths annually [1]. TB is most prevalent in the global South, and for which the response of respective governments and the international community is insufficient [2]. Yemen exhibits a comparatively low treatment success rate than the WHO’s target of 90% [3].TB is the fourth leading cause of death in Yemen [4]. Thus, high drug provisions and research are needed to develop novel solutions and approaches to combat the spread of TB in Yemen [5].

Health-related quality of life (HRQoL) is defined as “the extent to which patient’s subjective perception of physical, mental and social wellbeing are affected on a day to day basis by a disease and its treatment(s)” [6, 7]. Patients with chronic diseases highly value their mental and social wellbeing in addition to physical health [8]. The need to measure HRQoL has recently became important due to a broadening concept of measuring health status beyond conventional indicators, such as mortality and morbidity [9]. HRQoL is an indicator of the effect of diseases and associated morbidities on regular activities and functions. Consequently, HRQoL evaluation has become a crucial health outcome and an area of interest for policy makers, health care professionals and researchers. The HRQoL evaluation of patients with TB is imperative to identify appropriate actions for improving their health status and QoL [10]. Multidrug-resistant TB (MDR-TB) is caused by a strain of *Mycobacterium tuberculosis* (MTB) that is resistant to both isoniazid and rifampicin. MDR-TB is a chronic, devastating disease that requires protracted chemotherapy (≥ 20 months) with a severe regimen of potentially toxic and less efficacious second-line anti-TB drugs [11, 12]. MDR-TB has been described in an existing study as “the worst of the worst illnesses” and its treatment “worse than the disease itself” [13].

Despite the considerable attention given to traditional microbiological and clinical indicators, the effect of MDR-TB on the HRQoL of patients has largely been neglected [5]. Few studies have evaluated the HRQoL of MDR-TB patients. These studies have either assessed HRQoL cross-sectionally [14–16] or included small subsamples of MDR-TB patients among the drug-susceptible TB population [17]. Very few studies have longitudinally evaluated the effect of MDR-TB treatment on patients’ HRQoL [18]. A WHO’s progress report in 2015 on MDR-TB treatment remarked that “there is a dearth of literature about anti-TB drug-induced mortality, morbidity and loss in quality of life, particularly in low-resource settings” [2]. Therefore, this present study aims to evaluate the effect of MDR-TB and its treatment on the HRQoL of patients, particularly in Yemen. This study will help fill the gap of reported outcomes among MDR-TB patients and provide the much needed data concerning the effect of MDR-TB treatment on the HRQoL of patients. This study emphasizes on Yemen as it is among the 22 countries with an annual increase in MDR-TB cases and low health service based on latest WHO reports [19]. An estimated 1.7% patients with MDR-TB were detected recently among new TB patients and 15% among retreated patients in Yemen [19].

## Methods

### Study setting and population

This work is a prospective cohort study conducted in four among 5 main MDR TB centres in Yemen. These centers consider as the largest in Yemen. In addition all major diagnosis test such as culture is performed only in these centers. All eligible candidates (≥18 years of age, literate and can comprehend Arabic) were patients with MDR-TB enrolled for treatment at the study site and follow-up after 1 year of completing treatment. Those excluded comprised patients with a history of MDR-TB treatment and physical and/or cognitive limitations that prevent them from being able to answer questions. The treatment protocol of MDR-TB patients enrolled in this study has been previously reported [18].

### HRQoL assessment questionnaire

The eligible MDR-TB patients completed the SF-36 V2 survey at the beginning and end of treatment and after 1 year of follow up after completing the treatment. The short form SF-36 V2 health survey has been utilized by numerous TB studies and has shown acceptable reliability and validity [10, 20–22]. SF-36v2 consists of eight scales that measure eight domains of HRQoL, namely, physical functioning (PF, 10 items), role-physical (RP, four items), bodily pain (BP, two items), general health (GH, five items), vitality (VT, four items), social functioning (SF, two items) role-emotional (RE, three items) and mental health (MH, five items). These eight domains are categorized into two groups referred to as physical component summary (PCS) and mental component summary (MCS). PF, RP and BP scales are strongly correlated with PCS, whereas MH, RE and SF scales are correlated with MCS. VT and GH are moderately correlated with both PCS and MCS [23]. The SF36v2 standard form (one-month recall period) used in this study was issued by QualityMetric under the license number QM016042.

A pilot testing of SF-36v2 (Arabic translation) was performed on 38 eligible patients with MDR-TB, excluding those involved in the final study. All eight health domain scales displayed good internal consistency. The reliability coefficients ranged from 0.73–0.92, which were well within the acceptable limits (Cronbach’s α > 0.7, Nunnally, 1994). PCS exhibited strong correlation with PF, RP and BP (r ≥ 0.5) and moderate correlation with GH (r = 0.49). Similarly, MCS was strongly correlated with MH, RE and SF (r ≥ 0.5) and moderately correlated with VT (r = 0.47).

### Data collection

**Fig. 1:**
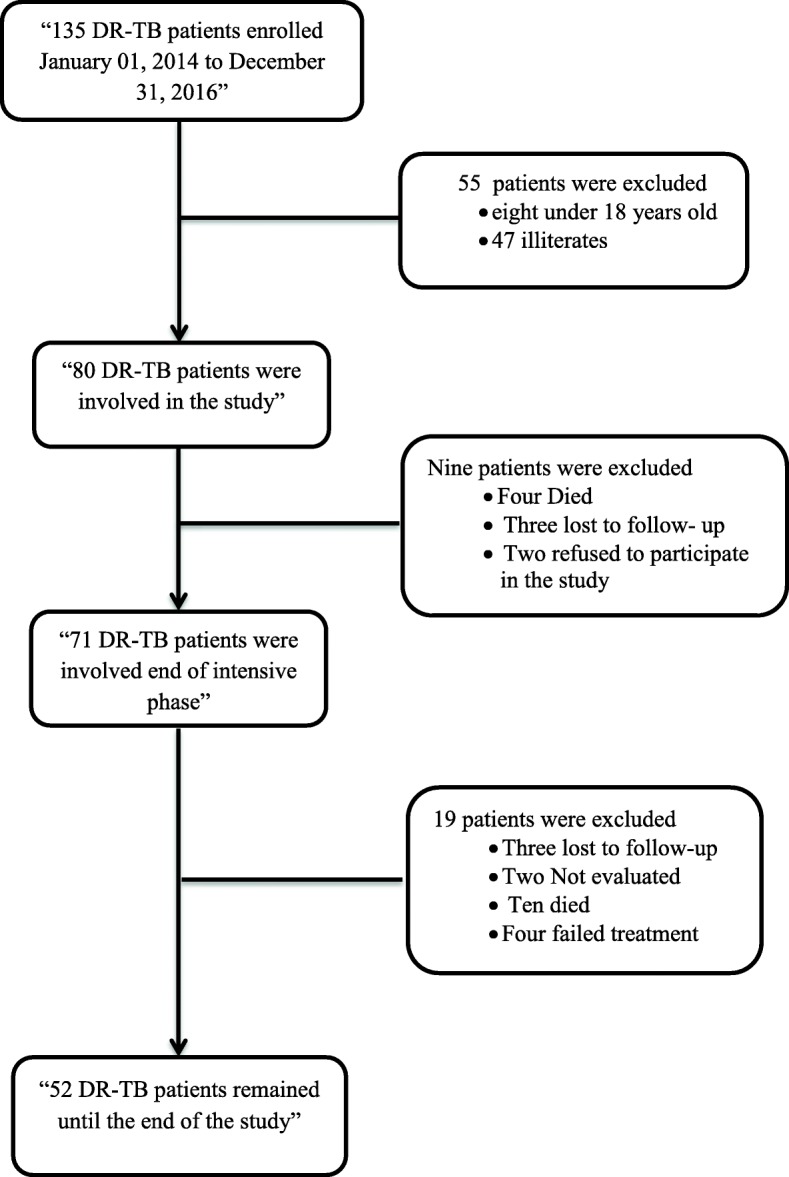
Number of drug-resistant tuberculosis patients involved in the study

During the study period, all eligible patients with MDR-TB who consented to participate in the study were requested to self-complete SF-36v2 at three time points: i) at beginning of treatment, ii) end of treatment and iii) 1 year follow-up after completing treatment. The registered subjects who did not participate at the second follow-up were not requested to complete the questionnaire on the third follow up. During the study recruitment period, 135 patients with MDR-TB were registered for the treatment at the study site. Fifty-five patients (41%) were ineligible (illiterates and < 18 years old) and were thus excluded. Eighty patients were eligible and completed the questionnaire at the baseline visit. Afterward, 71 and 52 patients completed the questionnaire on the second and final follow-ups, respectively (Fig. 1). Validated data collection forms were used to collect the socio-demographic and clinical data of the patients. Ethical approval for this study was obtained from the National Committee of Health (Ministry of Health-Sana’a/Yemen).

### Scoring

The QualityMetric Health Outcomes™ Scoring Software 4.5 was utilized to score the questionnaires. The standard norm-based scoring (NBS) of the eight health domains and summary components were calculated based on the standard scoring algorithms (US weights) recommended by the developers of the instrument [23] and by similar studies [22, 24–27]. A high SF-36 score indicates improved HRQoL outcome. During treatment, a variation of ≥3 NBS points in summary component measures and health domain scales denotes minimal important difference (MID) [23, 28]. A score range of 47 to 53 NBS points on component summary measures and health domain scales correspond to general population norms. A score of < 47 NBS points is suggestive of function impairment, whereas an individual is considered at risk of being depressed if he/she shows an MCS score of < 43 NBS points [22, 23]. To simplify the grading categories, we combined the four grades (i.e. scanty positive, 1+, 2+ and 3+) into two categories. Scanty positive and 1+ represented low-grade sputum, whereas 2+ and 3+ denoted high-grade sputum [22, 29, 30].

### Statistical analysis

Data were analyzed using SPSS 24. General linear model (GLM) repeated measures ANOVA was used to determine a possible significant interaction effect and variation in the pattern of subjects over time, as well as which independent variables were predictive of changes in summary scores. Following the detection of statistically significant interaction, Bonferroni post hoc test was applied to refine the interpretation. A *p*-value of < 0.05 was considered statistically significant. Cohen recommended frequently applied guidelines to rank the effect of size (small, partial eta squared = 0.01; moderate, partial eta squared = 0.06; large, partial eta squared = 0.14) [31].

## Result

### Patient clinical and socio-demographic characteristics

The baseline clinical and socio-demographic characteristics of the patients participating in the study are provided in Table 1. A total of 135 patients with MDR-TB were enrolled in the study, among which 80 (59.3%), 71 (52.6%) and 52 (38.5%) were in the beginning and end of treatment and at 1 year follow-up after completing treatment, respectively. Approximately 46% of the respondents were from Alhodidah, whereas the remaining respondents were evenly distributed among the other cities. As shown in Table 1, the majority of the patients with TB who registered in this study were male (60%), urban dwellers (56.3%), > 45 years old (60%), smokers (85%), unemployed (83.3%), khat chewers (91.3%), married (76.3%), with monthly income of ≤10,000 rial (83.8%) and with baseline weight of ≤40 kg (63.8%). The other characteristics are presented in Table 1.

**Table 1:** Socio-demographical characteristics of patients with MDR-TB (*n* = 80)

5.1.Characteristics	Patient *n* (%)
Governorates	
Alhodidah	37 (46.3)
Taiz	17 (21.3)
Sana’a	10 (12.5)
Aden	16 (20)
Gender	
Male	48 (60)
Female	32 (40)
Age (years)	
≤ 45	32 (40)
> 45	48 (60)
Baseline weight (kg)	
≤ 40	51 (63.8)
> 40	29 (36.3)
Area	
Rural	35 (43.8)
Urban	45 (56.3)
Marital status	
Married	61 (76.3)
Unmarried	19 (23.8)
Smoking	
Non-smoker	12 (15)
Smoker	68 (85)
Chewing khat^€^	
Yes	73 (91.3)
No	7 (8.8)
Employment status	
Employed	13 (16.3)
Unemployed	67 (83.3)
Monthly income (rial^¥^)	
≤ 10,000	67 (83.8)
> 10,000	13 (16.3)
History of TB^€^ treatment	
Previously treated	75 (93.8)
Newly treated	5 (6.3)
History of Streptomycin use	
No	57 (71.3)
Yes	23 (28.8)
Comorbidity	
No	54 (67.5)
Yes	23 (28.8)
Number of first line resistance	
≤ 2	55 (68.8)
> 2	25 (31.3)
Sputum grading	
Low	35 (43.7)
High	45 (56.3)
Baseline lung cavity	
No	39 (48.8)
Yes	41 (51.3)
Hb^¥^ level	
Normal	28 (35)
Below normal	52 (65)
Creatinine level	
Normal	71 (88.8)
Above normal	9 (11.3)
WBCs^α^ level	
Normal	43 (53.8)
Above normal	37 (46.3)
Bilirubin level	
Normal	71 (88.8)
Above normal	9 (11.3)

^**€**^khat: a shrub that grows in parts of East Africa and Yemen, **¥** rial: one dollar is approximately equivalent to 215 rial, αWBCs = white blood cells; ¥ Hb: haemoglobin

*Normal ranges: Hb = Male > 13 g/dl, Female > 11.5 g/dl; WBCs = > 11,000/ mm3; Creatinine = Male < 1.1 mg/dl, Female < .9 mg/dl; Bilirubin = ≤ 1 mg/dl, ^€^TB: tuberculosis;

### HRQoL domain scores in the beginning and end of treatment and at 1 year follow-up after treatment

Table 2 outlines the NBS of the eight health domain scales at various phases of MDR-TB treatment and after 1 year of completing treatment. Significant differences were observed in the mean scores for all HRQoL domains. At the beginning of treatment, the mean scores for all health domains were < 47 NBS points. At the completion of treatment, only PF and GH exceeded 47 NBS points. However, the score decreased after 1 year of completing treatment. Minimal important difference (MID), which is denoted by a change of ≥3 NBS points, was observed for all health domains between the beginning and end of treatment. MID was not observed for any health domain between the end of treatment and at 1 year follow-up after completing the treatment. The mean scores of component summary measures are presented in Table 3. The physical component scores (PCS) were different in the three treatment phases, that is, the scores at the beginning and end of treatment and at the 1 year follow-up after completing treatment were 36.9 ± 5.1, 44.3 ± 7.6 and 42.7 ± 7.8, respectively. The mental component scores (MCS) were different in the three treatment phases, that is, the scores at the beginning of treatment, continuation phase and at 1 year after completed treatment were 19.8 ± 4.4, 27.3 ± 8.1 and 26.7 ± 7.6, respectively. The other HRQoL subscale scores are shown in Table 2.

**Table 2:** Health-related quality of life SF 36 V2 scores using norm-based scoring (NBS) at various stages of treatment

Scale	Mean score (SD)		
	Beginning of treatment (*n* = 80)	End of treatment (*n* = 71)	1 year follow-up (*n* = 52)
PF*	40.7 (17.9)	59.3 (25.1)	51.5 (25.1)
RP ^ǂ^	16.1 (13.1)	31.1 (24.2)	30.6 (22.9)
BP‴	21.6 (16.1)	40.9 (23.5)	39.1 (22.6)
GH^¶^	28.3 (19.1)	48.5 (24.8)	47.8 (24.5)
VT ^ˠ^	14.55 (8.45)	30.5 (21.3)	30.2 (22.4)
SF^z^	25.9 (17.6)	46.6 (26.4)	43.7 (24.1)
RE^ʏ^	13.7 (13.1)	26.6 (22.6)	26.2 (21.1)
MH^€^	14.7 (11.3)	27.7 (18.7)	27.2 (15.8)

^**ʜ**^ NBS: Norm-based scores; *PF: Physical functioning; ^**ǂ**^RP: Role-physical; ‴BP: Bodily pain; GH: ^**¶**^General health; ^**ˠ**^ VT: Vitality; ^z^SF: Social functioning; ^**ʏ**^RE: Role-emotion; ^**€**^MH: Mental health

**Table 3:** Changes of physical and mental component summary (PCS and MCS) during various stages of treatment

Component Summary	Mean score (SD)			Alteration of Mean Score
	Start of treatment (*n* = 80)	End of treatment (*n* = 71)	1 year follow-up (*n* = 52)	
PCS*	36.9 (5.1)	44.3 (7.6)	42.7 (7.8)	7.4
MCS^ǂ^	19.8 (4.4)	27.3 (8.1)	26.7 (7.6)	7.5

*PCS: Physical component score; ^ǂ^MCS^:^ Mental component score

During the treatment, MID was observed for both PCS and MCS, although both scores remained < 47 NBS points on all settings. An increment in the PCS and MCS scores was observed the longer a patient remains under treatment. PCS was greater than MCS in both the beginning of treatment and after 1 year of completing treatment. However, MCS and PCS decreased after completing treatment. For both PCS and MCS scores, one-way repeated measure ANOVA revealed significant interaction effect for time (Table 4).

**Table 4:** General linear model (GLM) repeated measure ANOVA test for physical and mental component score within the subject effect

SOURCE	df	F	P-value	Partial eta squared
Physical Component Score				
Time*Gender	2	0.027	0.974	0.001
Time*Age	2	2.859	0.062	0.054
Time*baseline weight	2	0.456	0.635	0.009
Time*Smoking	2	0.668	0.515	0.013
Time*Employment	2	0.177	0.838	0.004
Time*Income (Rial)**°**	2	0.168	0.765	0.003
Time*Tuberculosis History	2	0.105	0.894	0.002
Time*History of Streptomycin use	2	1.772	0.175	0.034
Time* Comorbidity	2	2.941	0.057	0.056
Time* Number of first line drug resistance	2	1.008	0.369	0.20
Time* Sputum Grading	2	1.800	0.171	0.035
Time* Baseline Lung Cavity	2	1.219	0.300	0.024
Time* Stigma	2	0.121	0.886	0.002
Time* Residence	2	0.181	0.982	0.001
Time* Marital Status	2	1.141	0.322	0.022
Time* White Blood Cell Level	2	1.046	0.355	0.020
Time* Haemoglobin Level	2	0.055	0.947	0.001
Time* Bilirubin Level	2	0.541	0.584	0.011
Time* Chewing Khat ^**ǂ**^	2	0.736	0.482	0.014
Time* Length of sickness before MDR-TB diagnosis	2	3.534	**0.033**	0.066
Mental Component Score				
Time*Gender	2	0.295	0.745	0.006
Time*Age	2	28.553	**0.001**	0.363
Time*baseline weight	2	0.100	0.905	0.002
Time*Smoking	2	4.543	**0.013**	0.083
Time*Employment	2	0.413	0.663	0.008
Time*Income (Rial)**°**	2	0.412	0.543	0.005
Time*Tuberculosis History	2	0.025	0.975	0.001
Time*History of Streptomycin use	2	0.778	0.462	0.015
Time* Comorbidity	2	0.683	0.507	0.013
Time* Number of first line drug resistance	2	0.638	0.530	0.013
Time* Sputum Grading	2	0.151	0.723	0.003
Time* Baseline Lung Cavity	2	3.807	0.052	0.071
Time* Stigma	2	2.658	0.075	0.050
Time* Residence	2	4.013	**0.021**	0.074
Time* Marital Status	2	10.250	**0.001**	0.170
Time* White Blood Cell Level	2	0.448	0.640	0.009
Time* Haemoglobin Level	2	0.674	0.512	0.013
Time* Bilirubin Level	2	0.148	0.863	0.003
Time* Chewing Khat ^**ǂ**^	2	0.380	0.685	0.008
Time* Length of sickness before MDR-TB diagnosis	2	210.343	**0.001**	0.808

**°** Rial: Yemen’s currency; ^**ǂ**^ khat: Traditional Plant; *Greenhouse-Geisser values as sphericity cannot be assumed (*p* < 0.0005)

### Factors associated with HRQoL

Based on the results of GLM repeated measure ANOVA (Table 4), the length of sickness before DR-TB diagnosis was the only independent variable that interacted with time to predict trends in PCS scores within the subjects. However, age, smoking, residence, marital status and length of sickness before DR-TB diagnosis showed statistically significant interaction with time to predict trends in MCS scores.

**Fig. 2:**
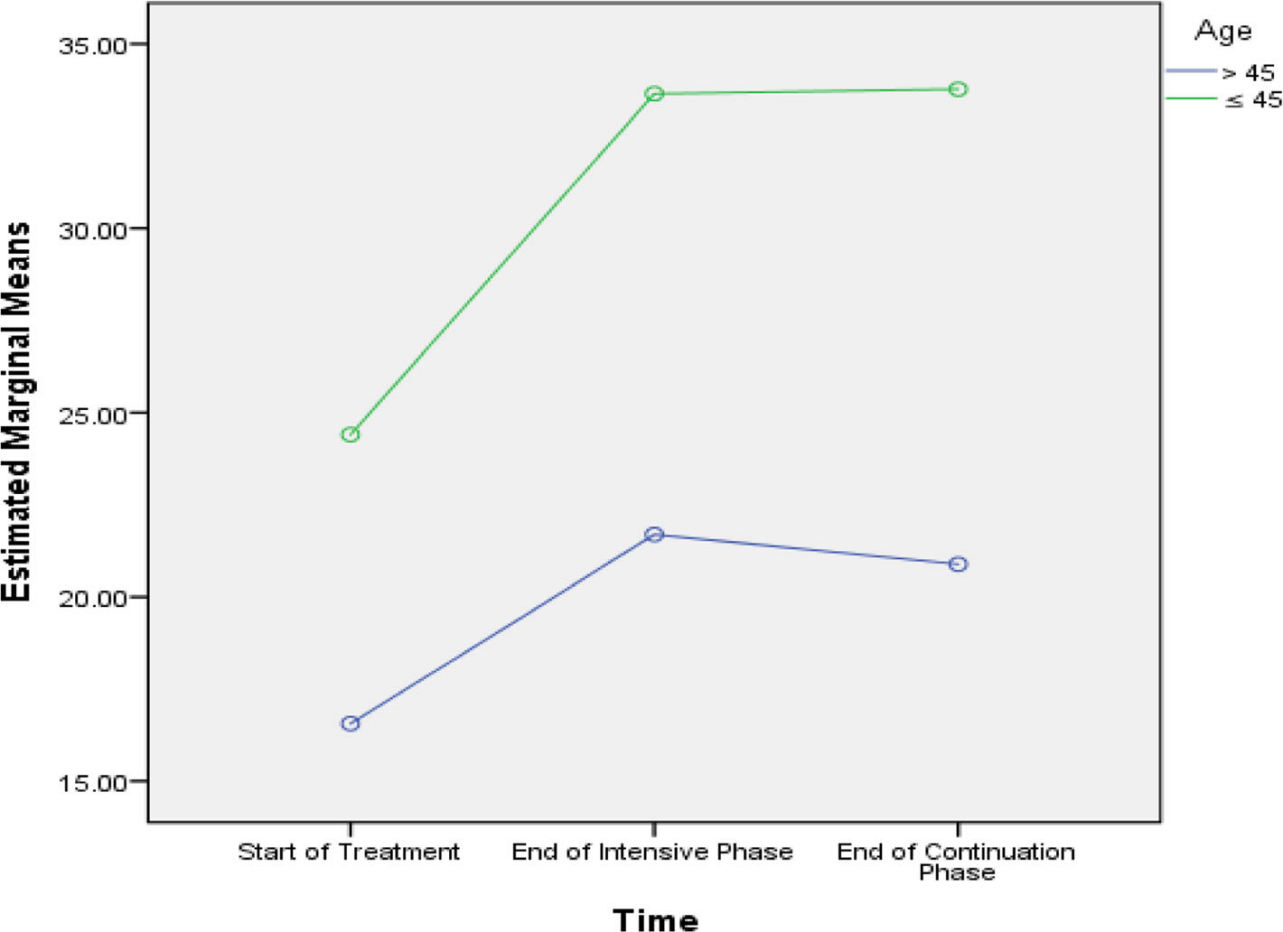
Age in years ≤45 and > 45: Difference in estimated marginal mean of MCS

**Fig. 3:**
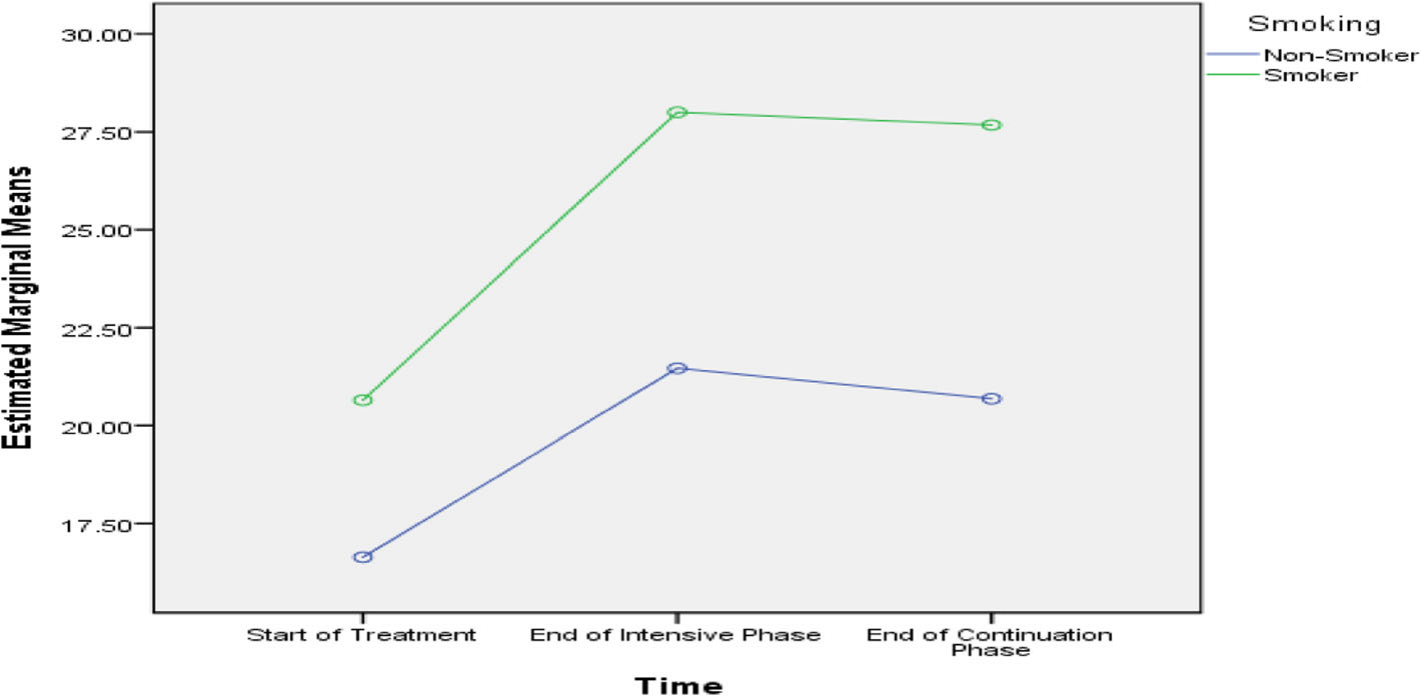
Smoker and non-smoker status: Difference in estimated marginal mean of MCS

**Fig. 4:**
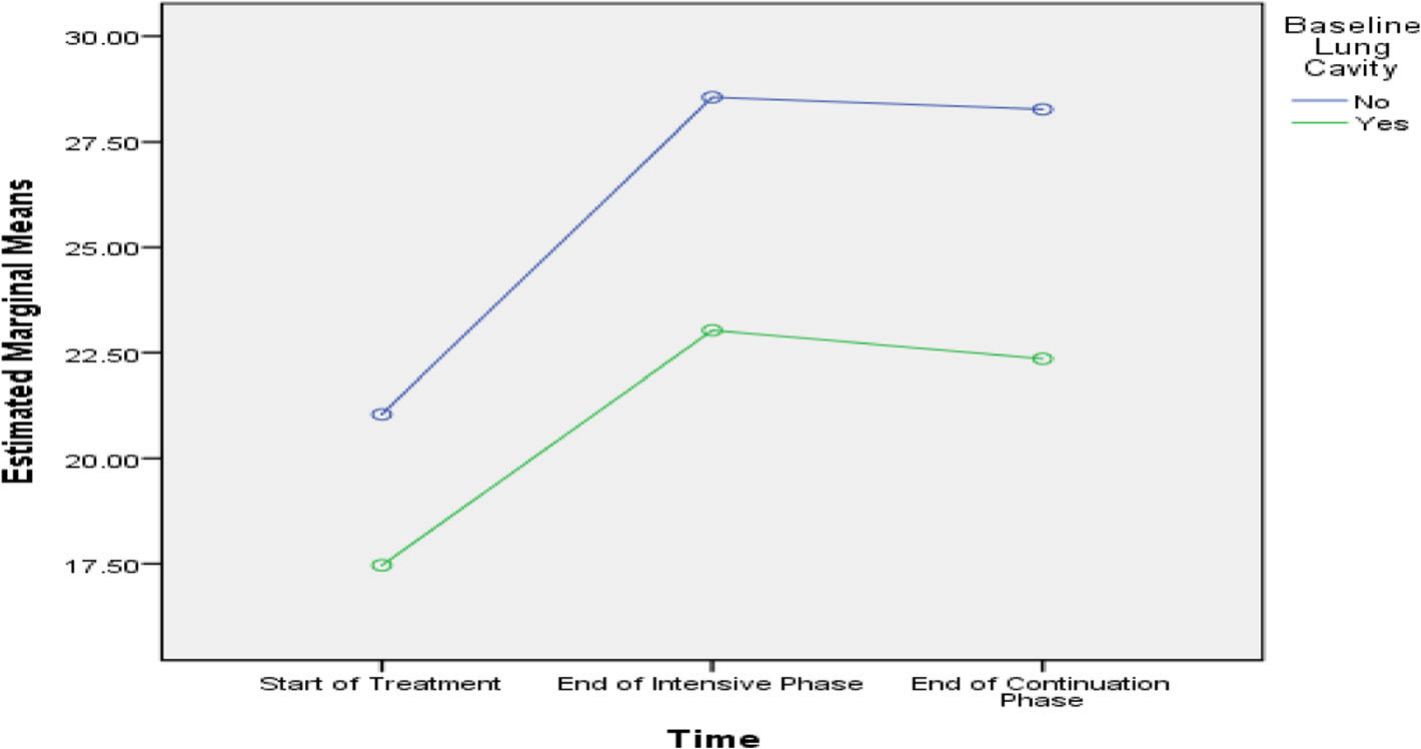
Presence and absence of baseline lung cavity: Difference in estimated marginal mean of MCS

**Fig. 5:**
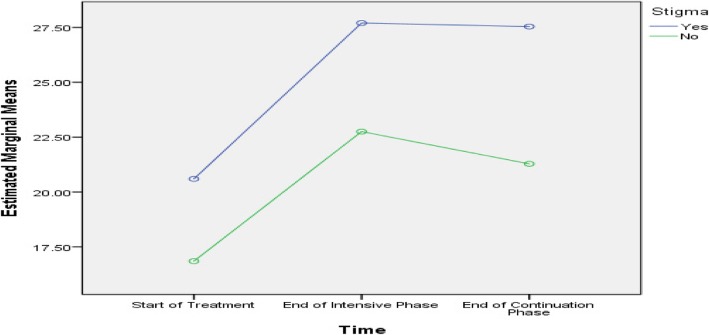
Stigma and non-stigma status: Difference in estimated marginal mean of MCS

**Fig. 6:**
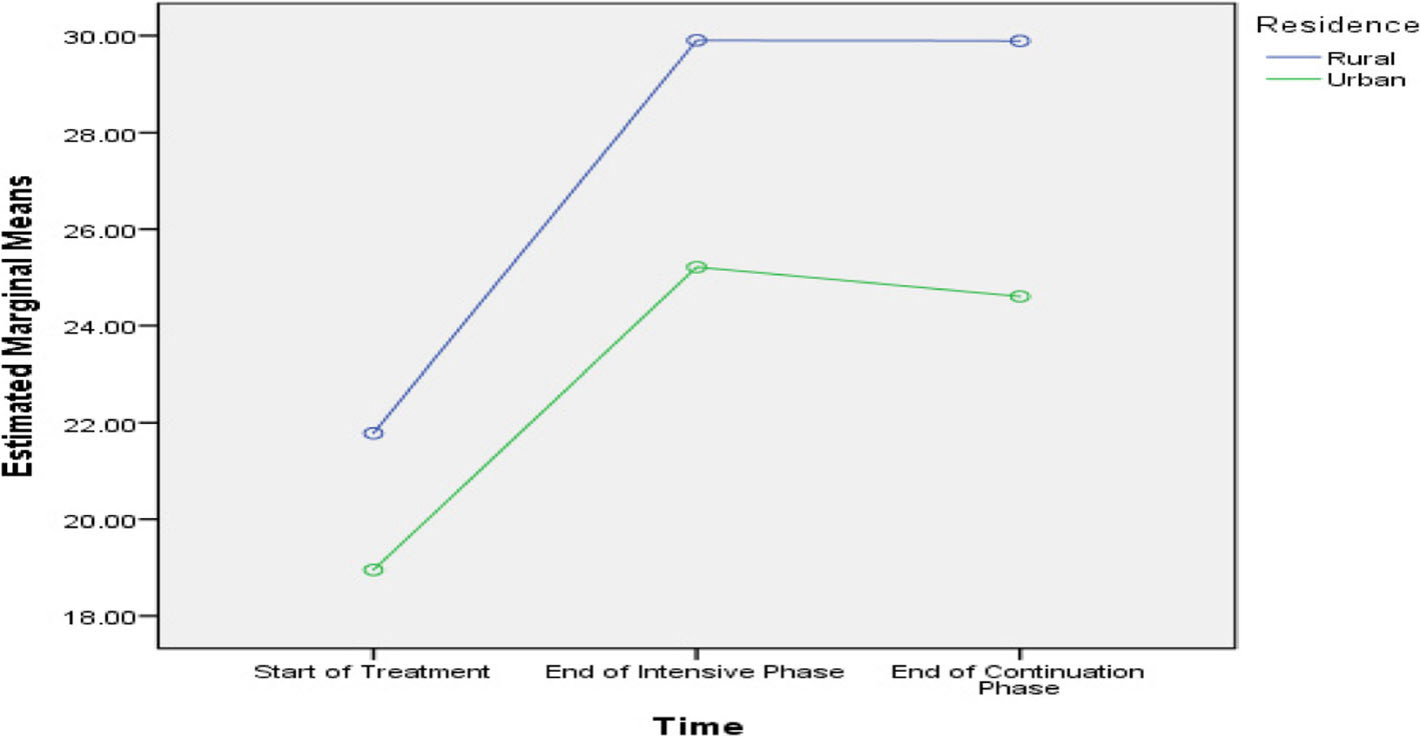
Rural and urban residence: Difference in estimated marginal mean of MCS

**Fig. 7:**
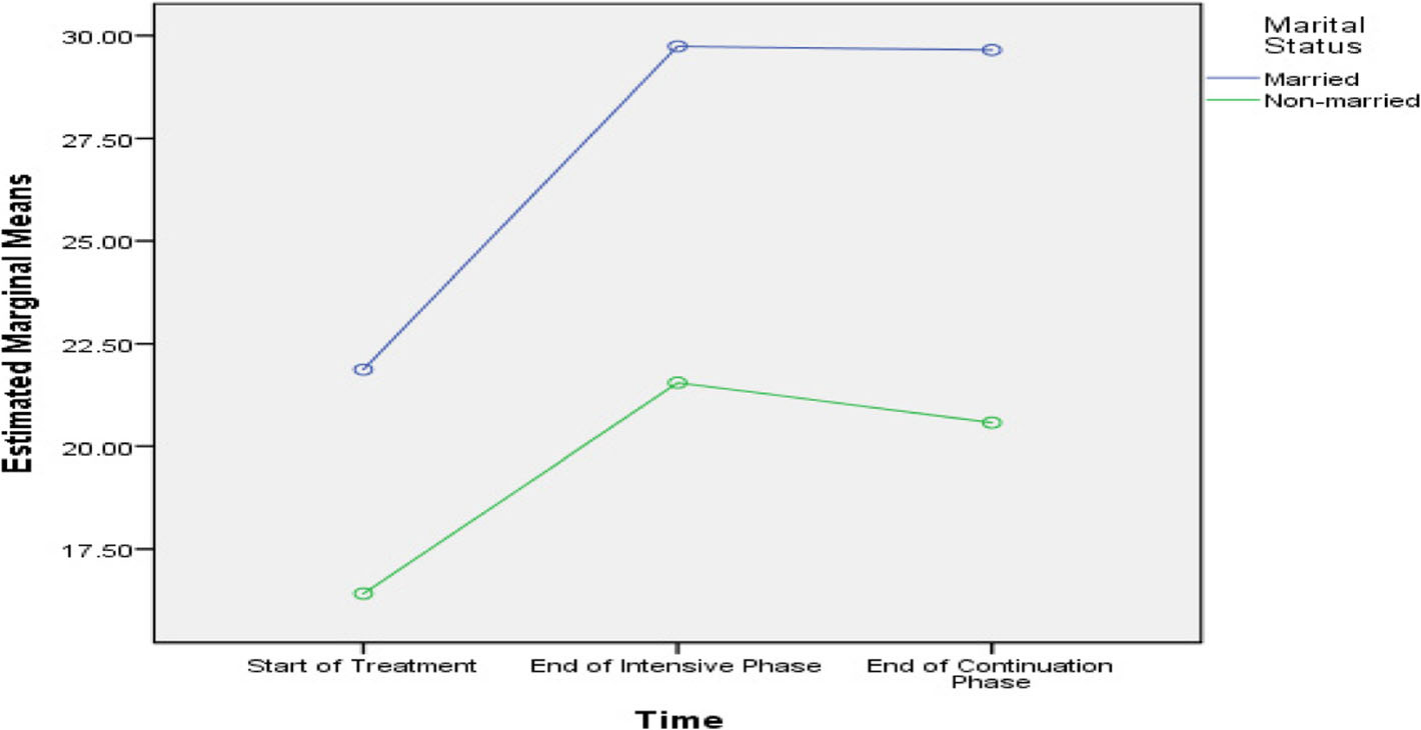
Married and unmarried status: Difference in estimated marginal mean of MCS

**Fig. 8:**
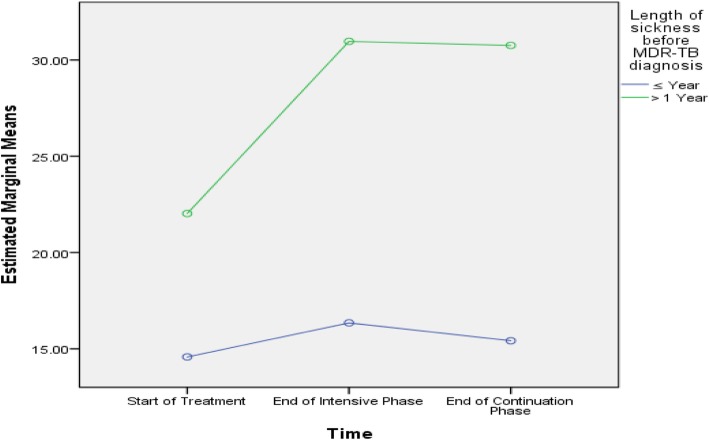
Length of sickness before MDR-TB diagnosis: Difference in estimated marginal mean of MCS

Table 5 shows that age, history of streptomycin use, baseline lung cavity, marital status and length of sickness before DR-TB diagnosis were predictive of difference in PCS scores among the subjects. Age, smoking, baseline lung cavity, stigma, residence, marital status and length of sickness before DR-TB diagnosis were predictive of difference in MCS scores among the subjects, as presented in Figs. 2, 3, 4, 5, 6, 7 and 8 respectively. As the difference between the groups at Times 2 and 3 were largely indicative of the difference observed at Time 1, the disparities in the PCS and MCS scores were possibly attributed to the differences in the composition of the groups.

**Table 5:** General linear model repeated measure ANOVA test for physical and mental score between the subject effect

SOURCE	df	F	P-value	Partial eta squared
Physical component score				
Time*Gender	1	1.553	0.219	0.030
Time*Age	1	7.402	**0.009**	0.129
Time*baseline weight	1	0.238	0.628	0.005
Time*Smoking	1	1.080	0.304	0.021
Time*Employment	1	0.042	0.838	0.001
Time*Income (Rial)**°**	1	0.032	0.786	0.002
Time*Tuberculosis History	1	0.881	0.352	0.017
Time*History of Streptomycin use	1	4.206	**0.046**	0.078
Time* Comorbidity	1	0.349	0.557	0.007
Time* Number of first line drug resistance	1	0.013	0.910	0.001
Time* Sputum Grading	1	0.983	0.326	0.019
Time* Baseline Lung Cavity	1	5.310	**0.025**	0.096
Time* Stigma	1	3.209	0.079	0.060
Time* Residence	1	1.197	0.279	0.023
Time* Marital Status	1	8.316	**0.006**	0.143
Time* White Blood Cell Level	1	0.704	0.405	0.014
Time* Haemoglobin Level	1	1.333	0.254	0.026
Time* Bilirubin Level	1	3.530	0.066	0.065
Time* Chewing Khat ^**ǂ**^	1	0.224	0.638	0.004
Time* Length of sickness before MDR-TB diagnosis	1	4.572	**0.037**	0.84
Mental component score				
Time*Gender	1	0.518	0.475	0.010
Time*Age	1	95.737	**0.001**	0.657
Time*baseline weight	1	0.563	0.457	0.11
Time*Smoking	1	6.843	**0.012**	0.120
Time*Employment	1	0.921	0.342	0.018
Time*Income (Rial)**°**	1	0.812	0.232	0.007
Time*Tuberculosis History	1	0.008	0.929	0.001
Time*History of Streptomycin use	1	1.135	0.292	0.022
Time* Comorbidity	1	0.012	0.993	0.002
Time* Number of first line drug resistance	1	0.328	0.570	0.007
Time* Sputum Grading	1	0.061	0.806	0.001
Time* Baseline Lung Cavity	1	7.139	**0.010**	0.125
Time* Stigma	1	4.787	**0.033**	0.087
Time* Residence	1	4.995	**0.030**	0.091
Time* Marital Status	1	21.585	**0.001**	0.302
Time* White Blood Cell Level	1	0.116	0.735	0.002
Time* Haemoglobin Level	1	0.150	0.700	0.003
Time* Bilirubin Level	1	0.053	0.820	0.001
Time* Chewing Khat ^**ǂ**^	1	0.513	0.477	0.010
Time* Length of sickness before MDR-TB diagnosis	1	130.69	**0.001**	0.723

**°** Rial: Yemen’s currency; ^**ǂ**^ khat: Traditional Plant; *Greenhouse-Geisser values as sphericity cannot be assumed (p < 0.0005)

## Discussion

HRQoL measurement adds a new perspective to the evaluation of TB programmes. Since the inception of effective treatment strategies where mortality is expected to be negligible, the emphasis of TB management has moved to the decrease in illness-related morbidity. In this context, this study highlights the post-treatment HRQoL of patients with TB registered in the MDR TB centres in Yemen as assessed by the extensively utilized generic measure of health status, the SF-36 V2 questionnaire. This study is the first to longitudinally evaluate the effect of MDR-TB treatment on patients’ HRQoL in Yemen. This study provides the much-sought data regarding the effect of MDR-TB treatment on patients’ HRQoL in Yemen. The HRQoL scores were generally higher in the late phases of TB treatment, which reflect the role of treatment in improving the quality of life of patients with TB. Follow-up patient in a duration of 1 year showed a significant decline in HRQoL scores reflecting the effect of the disease on patient, even after completing treatment. Increased attention should be considered by the Yemen Ministry of Health to improve the QoL after treatment by following up patients with MDRTB or implement any intervention programme to enhance the HRQoL.

Preliminary evaluation determined the participants’ scores of < 47 NBS for all health domains. However, two (i.e. PF and GH) of the eight health domain scales showed improved scores at the end of treatment. A low score, particularly on mental health, reflects the severely deteriorated condition of the patients. Similar poor HRQoL in patients with drug-susceptible and MDR-TB has been previously reported [10, 14, 15, 17, 20, 22, 32]. However, this current study could not attribute the severely deteriorated HRQoL to MDR-TB or prior TB episodes due to the absence of control group of patients with drug-susceptible TB. Upon initial evaluation, the RE, VT and MH of the study participants were observed to be the most affected health domains, indicating that the patients were mentally strained, possibly due to emotional stress. Thus, the overall mental health of the participants was considered poor with the fear of further deterioration [22]. Relatively lower mean score for MCS than that for PCS after treatment indicates increased mental distress and role limitation due to emotional complications rather than physical difficulties. Similarly, low scores on psychological health domain in patients with MDR and drug-susceptible TB have been reported in previous studies [15, 20, 22]. Therefore, a major focus of this study was on those factors that interact with time to predict the mental score either within and between the subjects.

The second evaluation at the end of treatment showed significant improvements in all health domain scales and both component summary measures. A plausible reason for the improvements between the two time points may be the positive effect of treatment. This finding is consistent with several studies on patients with drug-susceptible TB that reported MID at the end of treatment [21, 22, 32, 33]. This result was not unexpected, given that patients with MDR-TB are subjected to regular doses of multiple tablets and intramuscular injections of an extreme regimen during treatment [12]. The patients must adjust to the severe and painful side effects associated with the treatment phase, which is very challenging and uncomfortable. Interestingly, the participants of an earlier qualitative study described the MDR-TB after treatment as worse than the disease itself [13].

Notwithstanding clinically significant improvements, the HRQoL scores of the study participants at the end of TB treatment were less than the general population norms (47–53 NBS points), except for PF and GH. This finding is logical, as one may anticipate high prevalence of residual impairment in the HRQoL of these patients, given the lingering and destructive nature of MDR-TB and lengthy therapy with toxic drugs for 2 years. Similar residual impairments of HRQoL among patients with drug-susceptible TB have been reported in detailed reviews and specific studies [22, 20, 34]. Final evaluation showed that the MCS score of the patients was relatively lower than their PCS score, which is consistent with previous studies [15, 32].

Furthermore, this study identified a high prevalence of depression risk among the study participants. Preliminary assessment showed that a majority of the study participants are at risk of depression (MCS < 43 NBS points), though this proportion declined upon MDR-TB treatment. Nonetheless, a considerable proportion of the patients remain at risk of depression after 1 year of treatment. The high proportion of patients at risk of depression can be attributed to prior multiple treatment failures, knowledge of suffering from a debilitating form of TB and psychiatric side-effects related to the use of cycloserine, ethionamide and fluoroquinolones [35]. A high prevalence of depression has similarly been reported among patients with MDR-TB in related studies [21, 22, 35]. However, the prevalence of depression is relatively higher than those reported in similar studies because participants in the current study suffered from a deteriorating and lethal form of TB, were treated with drugs with known adverse psychiatric side-effects and indulge in an altered life style to adapt to the long course of MDR-TB painful therapy. Thus, the relatively higher prevalence of depression among patients with MDR-TB than that among patients with drug-susceptible TB at any stage of treatment is expected.

In this study, we determined the length of sickness before MDR-TB diagnosis interacted with time to predict trends in the PCS and MCS scores within the subject. Similarly, the general linear repeated model measure of ANOVA test showed that age, smoking, and rural residency and marital status interacted with time to predict the mental scores within the subject.

Based on the predictive of difference in MCS scores, mental health or well-being was significantly associated with smoking, age of > 45 years, baseline lungs cavity, stigma, residence, marital status and length of sickness. The non-smokers, patients aged ≤45 years, those with no baseline lung cavity, rural dwellers and non-stigmatised patients displayed higher MCS scores at every time point compared to their counterparts. These findings are consistent with some studies carried out in Canada and India [1, 26]. However, a study performed in China did not discover any relationship between smoking and MCS scores [10]. In general, smoking adversely affects the immune system, thus increasing the susceptibility of smokers to infections. Similarly, smokers tend to carry high bacillary loads due to compromised immune system. A high bacillary load can intensify the severity of disease, which may negatively affect the perception of the patients towards their mental and physical health. A prior study on the perceptions of TB patients determined at the end of treatment showed that social stigma persisted even after clinical and bacteriological cure and was similar in both males and females [36, 37].

The old age of > 45 years among patients was predictive of differences in overall MCS scores. Women are twice more likely than men to develop depression because of biological responses, self-concepts and coping mechanisms [38, 39], even when confronted with similar issues [38, 40]. Biologically, people with old age tend to exhibit dysregulated hypothalamic-pituitary-adrenal axis (HPA) response to stress, which increases their susceptibility to depression [41]. In developing countries like Yemen, a dearth of old age empowerment exists, which can further deteriorate the health, both physically and mentally, of those suffering from a chronic debilitating disease like MDR-TB.

Marital status interacted with time to be predictive of change in mean scores of component summary measures (PCS and MCS). The substantial increase in the mean MCS score of married patients during the first 12 months of treatment may be a result of the improved care and emotional support provided by the spouses and children of married patients [42]. Marriage has been extensively documented as a strong protective factor for depressive symptoms [43].

The length of sickness before DR-TB was the only independent variable that interacted with time to predict trends in PCS scores. The length of sickness also showed statistically significant interaction with time to predict trends in MCS scores and was predictive of difference in PCS and MCS scores. The relatively lengthy period of futility, social stigma, insufficient social support, sense of irrelevance, fear of disease and expected demise may have contributed to the relatively worse physical and mental health of the patients. The length of sickness and depression are positively associated among patients with TB [44].

The direct relationship between physical deterioration and low mental health [45] and the overproduction of interleukin-6 in chronic infections may be other plausible causes [46] of the significantly low mental health. The significantly low HRQoL among patients with long duration of sickness prior to the diagnosis of MDR-TB emphasizes the need for the early diagnosis of drug-resistant TB. A major reason for the long duration of sickness prior to the diagnosis of MDR-TB is the conventional practice of treating Category-I failures with Category-II regimen instead of assessing them for drug resistance. The efficacy of Category-II regimen for Category-I failures has been uncertain, particularly in the setting of an effective DOTS (Directly Observed Treatment, Short Course), where the majority of Category- I failures are associated with MDR-TB [47–49]. Given that Yemen is an MDR-TB high-burden country, Category-I failures must be evaluated for drug resistance according to WHO guidelines instead of placing them on Category-II regimen [42].

The provision of rapid drug susceptibility testing (Xpert MTB/RIF assay) will greatly decrease the delay in MDR-TB diagnosis, which in turn will prevent further reduction in patients’ HRQoL. Another reason for the prolonged duration of sickness with TB prior to the diagnosis of MDR-TB may be the delay in unreasonably searching for treatment centres by chest symptomatics, eventually reaching the chest clinic and being diagnosed with TB [18]. Ways that can effectively reduce the delay in diagnosis of drug-susceptible and -resistant TB include increasing public consciousness about TB symptoms, dangers of self-medication and availability of free diagnostic and treatment at public health facilities and developing effective collaborative endeavours between national tuberculosis control programme (NTP) and private health sectors [18, 50].

## Conclusion and recommendation

The length of sickness before DR-TB diagnosis interacted with time was found to be predictive of the trends in both PCS and MCS scores. However, age, smoking, residence, marital status and length of sickness before DR-TB diagnosis showed statistically significant interaction with time to predict trends in MCS scores. Despite the positive outcome of MDR-TB treatment, the poor HRQoL of study participants, particularly their mental health, at the end TB treatment demands the need for the urgent attention of NTP managers. Many of the patients face the risk of depression during MDR-TB treatment. The HRQoL data of patients with MDR-TB must be collected at the different stages of MDR-TB treatment to provide an additional parameter for assessing the effectiveness of the treatment programme. Patients with MDR-TB with known risk factors for poor HRQoL must be given special attention in the form of regular psychological counselling and material support (e.g. food rations and conveyance allowance). The psychological support can be provided to patients with MDR-TB through peer-to-peer and support groups, which may enable patients to meet and socialize with other patients and provide psychological support to each other. Inviting cured patients to support groups may provide emotional support for the patients on treatment.

### Limitations

The study was conducted in four main TB centres among all centres in Yemen. Thus, the findings must be interpreted with the major limitation of a small number of participating patients. Given that this study comprised patients from an extensive geographical area, the findings may reflect the effect of MDR-TB treatment on patients’ HRQoL across Yemen. Nonetheless, a multi-centre study with a large sample size and drug susceptible TB controls is needed to corroborate the findings. Given that the self-administered version of SF-36v2 health survey was used and the majority of the patients enrolled at the study site for treatment were illiterate, some were unable to fully participate in the study.

## Data Availability

The datasets used in this study are available from the corresponding author upon reasonable request.

## Abbreviations

BP: Bodily Pain
CP: Continuation Phase
GH: General Health
HPA: Hypothalamic-Pituitary-Adrenal
HRQoL: Health-Related Quality of Life
INH: Isoniazid
IP: Initial Phase
MCS: Mental Component Summary
MDR: Multi-Drug Resistance
MH: Mental Health
MID: Minimal important Difference
NBS: Normal Base Score
NTP: National Tuberculosis Program
PCS: Physical Component Summary
PF: Physical Functioning
RE: Role-Emotional
RMP: Rifampicin
RP: Role-Physical
SF: Social Functioning
TB: Tuberculosis
VT: Vitality
